# Applications of Transcriptomics and Proteomics for Understanding Dormancy and Resuscitation in *Mycobacterium tuberculosis*

**DOI:** 10.3389/fmicb.2021.642487

**Published:** 2021-03-31

**Authors:** Manikuntala Kundu, Joyoti Basu

**Affiliations:** Department of Chemistry, Bose Institute, Kolkata, India

**Keywords:** dormancy, resuscitation, *Mycobacterium tuberculosis*, transcriptomics, proteomics

## Abstract

*Mycobacterium tuberculosis* can survive within its host for extended periods of time without any clinical symptoms of disease and reactivate when the immune system is weakened. A detailed understanding of how *M. tuberculosis* enters into and exits out of dormancy, is necessary in order to develop new strategies for tackling tuberculosis. Omics methodologies are unsupervised and unbiased to any hypothesis, making them useful tools for the discovery of new drug targets. This review summarizes the findings of transcriptomic and proteomic approaches toward understanding dormancy and reactivation of *M. tuberculosis*. Within the granuloma of latently infected individuals, the bacteria are dormant, with a marked slowdown of growth, division and metabolism. *In vitro* models have attempted to simulate these features by subjecting the bacterium to hypoxia, nutrient starvation, potassium depletion, growth in the presence of vitamin C, or growth in the presence of long-chain fatty acids. The striking feature of all the models is the upregulation of the DosR regulon, which includes the transcriptional regulator Rv0081, one of the central hubs of dormancy. Also upregulated are chaperone proteins, fatty acid and cholesterol degrading enzymes, the sigma factors SigE and SigB, enzymes of the glyoxylate and the methylcitrate cycle, the Clp proteases and the transcriptional regulator ClgR. Further, there is increased expression of genes involved in mycobactin synthesis, fatty acid degradation, the glyoxylate shunt and gluconeogenesis, in granulomas formed *in vitro* from peripheral blood mononuclear cells from latently infected individuals compared to naïve individuals. Genes linked to aerobic respiration, replication, transcription, translation and cell division, are downregulated during dormancy *in vitro*, but upregulated during reactivation. Resuscitation *in vitro* is associated with upregulation of genes linked to the synthesis of mycolic acids, phthiocerol mycocerosate (PDIM) and sulfolipids; ribosome biosynthesis, replication, transcription and translation, cell division, and genes encoding the five resuscitation promoting factors (Rpfs). The expression of proteases, transposases and insertion sequences, suggests genome reorganization during reactivation.

## Introduction

Tuberculosis (TB) remains a global health problem with 1,000,000 new cases and 1,400,000 deaths in 2019 (World Health Organization, 2020). Drug resistance continues to be on the rise, with 465,000 reported cases in 2019. In the last 40 years, only three new drugs have been marketed for the treatment of TB, namely pretomanid, delamanid, and bedaquiline. This confirms the need for renewed efforts aimed at better understanding strategies for developing new chemotherapeutic agents as well as efficacious vaccines.

Latent TB is characterized by a positive tuberculin skin test without any symptoms of disease (Lordi and Reichman, 1988; Huebner et al., 1993). Houben and Dodd (2016) estimated in 2016 that out of the 1.7 billion people latently infected with *Mycobacterium tuberculosis*, 56 million could potentially reactivate into active disease upon weakening of the immune system. Dormancy is characterized by a reversible metabolic slowdown of the bacterium (Lewis, 2010). Kussell and Leibler (2005) and Kussell et al. (2005) show that even before sensing stress, a small fraction of the bacteria are in a non-growing state. The existence of a small subpopulation of cells with reduced metabolic activity, results in a given treatment (directed against growing cells) failing to kill this subpopulation giving rise to persisters, which can then resuscitate many years later. The molecular basis of latent TB infection remains incompletely understood. The shift to a dormant state is associated with transcriptomic and proteomic changes that reduce metabolic activity, increase the resistance of *M. tuberculosis* to environmental stresses and increase antibiotic tolerance (Caño-Muñiz et al., 2018). Once the stress is removed, dormant cells reactivate and replication is initiated. Treating the dormant state of *M. tuberculosis* requires a detailed understanding of the key processes and pathways that are adjusted when the bacteria transition into dormancy. It is equally important to understand the triggers of resuscitation so that these may be also be targeted.

Omics technologies free scientists from the biases of reductionist approaches. The unsupervised nature of omics technologies can open up new approaches for drug and vaccine development. Here we will discuss how omics technologies have been employed to understand dormancy and resuscitation of *M. tuberculosis*, focusing on transcriptomics and proteomics. We will first outline the models used for studying dormancy and resuscitation of *M. tuberculosis*, and then discuss the pathways, processes and targets which are differentially regulated in response to a shift toward dormancy and an exit out of dormancy. We will conclude by attempting to bring into perspective the implications of these dynamic shifts in gene and protein expression during the course of infection, our gaps in understanding of these shifts and how this knowledge may help in the combat of disease.

## The Granuloma in Tuberculosis

During the course of human pulmonary tuberculosis, inhaled *M. tuberculosis* is transported across the alveolar epithelium, where the formation of granulomas is initiated (Ramakrishnan, 2012). It was widely held that the host attempts to control infection by segregating the bacteria in the granuloma. However, it is now established that the bacterium itself plays an active role in granuloma formation (Co et al., 2004; Saunders and Britton, 2007; Paige and Bishai, 2010). Elegant studies using zebrafish infection with *M. marinum*, have established that the 6 kDa early secretory antigenic target (ESAT-6) induces the production of matrix metalloproteinase-9 (MMP-9), activation of the epithelium, and recruitment of macrophages to the site of infection (Volkman et al., 2010). The granuloma also enables the dissemination of infected macrophages to new sites (Davis and Ramakrishnan, 2009). This is accomplished when *M. tuberculosis* spreads from dying macrophages into newly recruited ones. Initially, the granuloma is a loose aggregate composed of recruited innate immune cells such as macrophages and neutrophils. This is followed by the recruitment of antigen-specific T lymphocytes, and the activation of infected macrophages. The hallmark of the granuloma is a central core containing infected macrophages surrounded by foamy macrophages, epitheloid cells and T lymphocytes. It is the niche in which dormant bacilli reside for extended periods of time and are associated with latent infection. As the infection progresses to active disease, a caseous core forms, consisting of dead or dying macrophages with extracellular *M. tuberculosis.* The bacilli within the core of the granuloma in latent TB reside within a hypoxic environment in which lipids are the major source of nutrients. The caseating granuloma presents a less hypoxic core than that associated with latent disease.

Evidence of hypoxia in the core of the granuloma has been demonstrated in pulmonary granulomas in mouse, guinea pig, rabbits, and non-human primates using pimonidazole hydrochloride, an imaging agent that is bioreductively activated only under hypoxic conditions. Hypoxia has been confirmed by directly measuring oxygen partial pressure with a fiber optic oxygen probe inserted into the granuloma (Via et al., 2008). The lipid-rich environment contains cholesteryl ester, triacylglycerides, and lactosylceramide (Kim et al., 2010). The transcriptional signature of *M. tuberculosis* RNA isolated from the lungs of chronically infected mouse, is reminiscent of the signatures associated with environmental conditions such as low pH, oxygen depletion, iron limitation, nitrosative stress and nutrient starvation, suggesting that these conditions are probably associated with bacteria residing in granulomas (Timm et al., 2003). In summary, long-term residence of *M. tuberculosis* within the granuloma, requires that the bacterium be able to adapt to oxygen-limited conditions and a lipid-rich environment that is likely limited in terms of availability of other nutrients and metal ions, and a high concentration of nitric oxide (Nathan and Ehrt, 2004). *In vitro* or *ex vivo* models for studying dormancy, have therefore attempted to recreate some of these *in vivo* conditions that the bacterium must encounter over the duration of its residence within the host.

## Models for Studying Dormancy and Reactivation

### *In vitro* Models

Hypoxia, a defining characteristic of the granuloma, is one of the most widely documented conditions that is associated with the transition of *M. tuberculosis* to a dormant state. As *M. tuberculosis* senses a gradient of oxygen depletion, it prepares itself for transition into a metabolically dormant, non-replicating state, known as non-replicating persistence (NRP). The most widely used model for studying NRP is the *in vitro* Wayne model (Wayne and Hayes, 1996). Wayne (1976) observed that as the concentration of dissolved oxygen (DO) decreases, *M. tuberculosis* arrests growth over extended periods of time but retains the ability to continue exponential growth once the DO concentration rises. Wayne’s model attempts to simulate the gradual depletion of oxygen in the granuloma. Bacteria are grown in sealed containers with a controlled ratio of air to culture medium equal to 0.5 (called the head space ratio or HSR). The model defines two states. The first termed NRP1 occurs as the oxygen saturation reaches 1%. The bacteria can no longer replicate but still have high levels of ATP (Wayne and Sohaskey, 2001). At NRP2, the oxygen saturation falls below 0.06%. As oxygen depletes, the bacilli adjust metabolism and enter growth arrest (Boshoff and Barry, 2005). The Wayne model is restricted by the fact that it fails to account for conditions other than hypoxia, that prevail in the granuloma and likely have an effect on bacterial metabolism.

Bacon et al. (2004) have employed a chemostat model in which aerobic cultures [dissolved oxygen tension (DOT) of 50%] were transferred to new vessels and allowed to stabilize to a DOT of 20% for 2–3 days. Low-oxygen cultures were established by lowering the DOT from 20 to 1% (hypoxic) in a stepwise manner over a period of 5–7 days, and allowed to reach steady state growth (i.e., a constant turbidity) before collection for gene expression.

Peterson et al. (2020) have employed a more accurate technique for generating an oxygen gradient. A programmable multiplexed reactor system has been designed to precisely monitor oxygen levels within the growth media, with minimal disturbance to the bacteria. Air and nitrogen gas lines were connected to separate mass flow controllers allowing programmable gradients of gas mixtures to be streamlined into the headspace of spinner flasks containing *M. tuberculosis* cultures. The DO contents of the cultures were measured using non-invasive fiber-optic technology. The reproducibility of this method is superior to that of the Wayne model and dissolved oxygen concentrations can be measured in real time.

Other *in vitro* models have been developed in attempts to simulate the conditions prevailing within the granuloma. Betts et al. (2002) established a model in which *M. tuberculosis* was subjected to nutrient starvation, by suspending cells in phosphate-buffered saline (PBS) for extended periods of time. Under these conditions, the bacterium underwent growth arrest and a decrease in respiration rate. Salina et al. (2014, 2019) have studied growth in potassium-deficient media which generates non-culturable (NC) bacilli, tolerant to cell wall targeting antimicrobials. Potassium supplementation enables resuscitation of growth. Rodríguez et al. (2014) have analyzed adaptation of *M. tuberculosis* to long-chain fatty acids (the main energy source of *M*. *tuberculosis* within the host milieu). Taneja et al. (2010) have reported that vitamin C triggers growth arrest and a dormancy phenotype in *M. tuberculosis*.

### The *in vitro* Granuloma Model

Granulomas contain macrophages, epitheloid cells and multinucleated giant cells surrounded by lymphocytes. Granulomas contain *M. tuberculosis* in a niche that is purported to be hypoxic. Conditions within the granuloma are intimately linked to the dormant state of *M. tuberculosis*. Guirado et al. (2015) have developed an *in vitro* granuloma model derived from human peripheral blood mononuclear cells from individuals with or without latent TB infection (LTBI).

### *In vivo* Models

Several *in vivo* models have been used to understand *M. tuberculosis* dormancy and reactivation using animals such as mice (Dutta and Karakousis, 2014), guinea pigs (Ordway et al., 2010), rabbits (Manabe et al., 2008; Subbian et al., 2012) and non-human primates (Kaushal et al., 2012; Peña and Ho, 2015). However, each model has its own drawbacks, most significantly in terms of the ability to reproduce the human disease pathology, the composition of the granuloma and the necrosis that is the hallmark of pathological tuberculosis. In the light of these limitations, *in vitro* models attempting to simulate the conditions of latency and reactivation, remain in vogue.

## Genes/Proteins Differentially Regulated During Dormancy

### Two Component Systems: The DosR Regulon

Out of the 190 regulatory proteins encoded by *M. tuberculosis*, 11 form the paired two-component systems (TCSs). In a typical TCS, a membrane-bound sensor kinase senses the environment signal which is subsequently transmitted through a phosphorelay to a cytoplasmic response regulator which regulates a subset of genes. These TCSs respond to environmental cues such as inorganic phosphate, SDS, oxygen, pH and nutrient limitation (Kundu, 2018). Of the paired TCSs, the DosR/DosS TCS is best characterized as the responder to hypoxia in the mycobacterial environment. The genes Rv3132c and Rv3133c encode a 578 amino acid histidine kinase protein (termed DosS) and a 217 amino acid response regulator protein (termed DosR). The signature feature of hypoxia-associated dormancy, is the upregulation of DosR and its regulon. Transcriptome analysis showed that *dosR* itself, and a *dosR*-regulated cluster of genes is induced early during hypoxia (Bacon et al., 2004; Muttucumaru et al., 2004; Voskuil et al., 2004; Iona et al., 2016). A partial list of *dosR*-dependent genes induced under hypoxia is given in Table 1. Intriguingly, Del Portillo et al. (2019) have reported that there are no genes in common between the *dosR* regulon and NRP1 during growth in fatty acid-containing medium. Under hypoxia, *M. tuberculosis* induces reduction of nitrate (NO_3_^–^) to nitrite (NO_2_^–^) to control redox homeostasis and energy production (Sohaskey and Wayne, 2003). The mycobacterial nitrate reductase (encoded by *narGHIJ*) as well as *narK2*, a nitrate transporter and *narX* (nitrate-reductase-like protein), members of the DosR regulon, are induced under hypoxia. These facilitate accumulation of nitrite under hypoxic conditions. In harmony with *in vitro* transcriptome and proteome analysis, transcripts from *narG* and *narX*, have been identified within granulomas of human TB samples (Rachman et al., 2006). Proteome analysis has also confirmed the induction of DosR during NRP1 (Gopinath et al., 2015) (Table 2). The induction of DosR was also evident in other models of dormancy, such as vitamin C exposure (Nandi et al., 2019).

**TABLE 1:** *Mycobacterium tuberculosis* genes upregulated in various *in vitro* models of dormancy.

Model	Method for achieving dormancy	Selected genes upregulated under hypoxia	References
Hypoxia	Wayne model	Dormancy regulon (day 4 of hypoxia). Ribosomal protein encoding genes *rpsR2*, *rpsN2*, and *rpmG1* (induced early during hypoxia, but repressed after day 20). Cytochrome bd oxidase (*cydA, cydB, cydC*, and *cydD*), induced early during hypoxia; *narX-narK2*; the iron storage bacterioferritin *bfrB* and mycobactin synthesizing genes; *nrdZ*, *ctpF*, *otsB*. Sigma factors: *sigB* (10–30 days of hypoxia); *sigE* and *sigC:* induced early during hypoxia, fall off at day 10 and day 12 respectively; *sigH*: induced throughout hypoxia.	Voskuil et al., 2004
Hypoxia	Chemostat culture under controlled oxygen tension. 50% dissolved oxygen tension (DOT): aerobic; 1% DOT: hypoxic	33 of the genes of the *dosR* regulon including the clusters *Rv0079* to *Rv0080* and *Rv0081* to *Rv0087, Rv0569*, *Rv0573c*, *Rv0575c*, *narX-narK2*, *Rv1996*, *Rv1997*. DosR-independent cluster consisting of *Rv2028c–pfkB–Rv2030c–acr–acg*, six genes of the mycobactin synthesis cluster (*Rv2377c to Rv2386c, mbtA-I*), *bfrB*; *Rv3182*, *Rv3183*, *Rv1964*, *Rv1130* (likely to encode 2-methylcitrate hydratase *prpD*) and *accD2* (predicted acetyl/propionyl CoA carboxylase).	Bacon et al., 2004
Hypoxia	Wayne model of dormancy; aerobic (in roller bottles), microaerophilic (NRP1, 1% oxygen) and anaerobic (NRP2, 0.06%) cultures.	Upregulated to a greater extent in NRP2 than in NRP1: Regulatory proteins *Rv3574 (KstR)*, *Rv2745c (ClgR)*, *Rv3833*, *Rv3334*, *Rv3291c* (leucine responsive regulatory protein, regulator for the leucine operon). *Rv1471* (thioredoxin) *Rv1997* (*CtpF*), cation transporting ATPase, *narK2*	Muttucumaru et al., 2004
Hypoxia	Wayne model, gene expression analyzed at different time points of hypoxia	*narK2* (upregulated from day 9 to day 40); *Rv2031c* (*acr*), *Rv3130c* (*tgs1*), *sigB*, *sigE*, *sigH*, *Rv1471* (*trxB1*), *Rv2454c* (2-oxoglutarate oxidoreductase, beta subunit), *Rv2455c* (2-oxoglutarate oxidoreductase, alfa subunit)	Iona et al., 2016
Hypoxia	Real time monitoring of oxygen levels in a programmable reactor system	*Rv0081*, *Rv3597c (Lsr2)*, *Rv1990c*, *Rv2034*, *Rv0023*	Peterson et al., 2020
Growth in the presence of even length long chain fatty acids	Cells were grown in dextrose to exponential (DE) or stationary (DS); or in the presence of even length long chain fatty acids to exponential (FE) or stationary (FS) phase. RNA was analyzed by ss-RNA-seq.	Overexpression of genes in FS over DS: tRNAs; *pckA*, *tgs1*, *icl1*; transcriptional regulators *whiB3*, *dosR*, *Rv0081*, *nrdR.* Non-coding RNA MTS2823	Rodríguez et al., 2014
Hypoxia in cultures supplemented with a mix of even long-chain fatty acids or dextrose as carbon sources	Cells were grown in Dubos medium supplemented with either: 0.2% dextrose (D), or long chain fatty acids (F) at a final concentration of 0.001% as main carbon sources. Exponential phase cultures at an OD_600_ = 0.4 were subjected to hypoxia according to the Wayne model.	Genes upregulated in D-NRP1 and F-NRP1*: Rv0251c*, *Rv1221*, *Rv2050*, *Rv2694c*, and *Rv2745c*, encoding the heat shock protein Hsp, the alternative sigma factor SigE, the RNA polymerase-binding protein RbpA, the conserved protein Rv2694c, TA modules *vapB10*, *vapC37*. *vapC2*0; and the transcriptional regulatory protein ClgR, respectively. Genes upregulated specifically in D-NRP1: *Rv0081*, *SigH*. Genes upregulated specifically in F-NRP1: *Rv3765c* (*trcX*). Rv1985c (*iciA*) expressed in D-NRP1 and F-NRP2. Non-coding RNA MTS2823	Del Portillo et al., 2019
Hypoxia in the presence of lipids as carbon source	Cells were grown in the presence of long chain fatty acids (C16:0, C18:0, and C18:1) and cholesterol, or dextrose as carbon source, and gene expression was analyzed in exponential phase, stationary phase, NRP1 and NRP2.	Genes upregulated during hypoxia in the presence of lipids: *Rv3161c, Rv3160c, Rv0678, Rv1217c, PPE53*, and *che1* (probable ferrochelatase), TA modules *vapB9/vapC9* and *vapB22/vapC22.*Non-coding RNA MTS2823.	Aguilar-Ayala et al., 2017
Vitamin C-induced dormancy	Cultures were diluted to OD_595_ ∼0.1 in Dubos medium (without ADC), Vitamin C was added and the tubes were incubated under shaking conditions.	Sigma factors *sigB*, *sigH*, *sigE*, *sigF*, *sigM*. TCS components *narL*, *tcrA*; transcriptional regulators *whiB1*, *whiB3*, *Rv0081, lsr2*; chaperone-encoding genes such as *hspX*, *dnaK*, *dnaJ1*, *grpE*, *clpB*, *hsp* (or *acr2*); proton pumping NADH dehydrogenase (*nuoA-G*; *nuoH-N*); *trxB1* (encoding thioredoxin), *icl* (encoding isocitrate lyase, a key enzyme of the glyoxylate pathway), *mymA* and *fadD13* genes of the *mymA* operon (Rv3083–3089), (involved in mycolic acid biosynthesis); *fadE5*, *fadE13* and *fadD19* (involved in fatty acid degradation); *fadE6*, *fadE28* and *fadE32* (putative acyl-CoA dehydrogenases); *scoA and scoB* (involved in the utilization of ketones); *pks1-papA1* (involved in sulfolipid synthesis), and *tgs1* (involved in triglyceride synthesis). Metal-ion transporter-coding genes, *ctpV* and *ctpG*; and *espA/Rv3616c* of the *Rv3616c–Rv3614c* operon that encodes the ESX-1 protein secretion system; antioxidant genes *furA*, *katG*, *glbN*, and *ahpC*.	Taneja et al., 2010; Sikri et al., 2015; Nandi et al., 2019
Nutrient starvation	Cultures were grown for 7 days in nutrient-rich media, cells were pelleted, then resuspended in PBS and left standing at 37°C in sealed bottles upto 6 weeks	*pdhABC* (subunits of the pyruvate dehydrogenase enzyme complex), *frdABCD* (the fumarate reductase complex), sigma factors (*sigB*, *sigE*, *sigF*, and *sigD*), regulatory genes *Rv2034*, *Rv1152*, *Rv3291c*, *whiB2*; the two-component system *kdpDE*, *subI–csyT–cysW–cysA* (sulfate transporters), the stringent response regulator *relA*	Betts et al., 2002
Potassium depletion	Cultures (OD_600_ 0.35–0.4) were inoculated into complete Sauton or potassium-deficient Sauton medium supplemented with ADC and Tween 80 and shaken at 200 r.p.m. for 39–41 days.	TCS components *mprAB*, *kdpD*, *prrA*; *whiB1*, *whiB6*; *kstR* (involved in cholesterol degradation pathway); *icl1*, *mutA* (methylmalonyl pathway). *bkdA*, *bkdB*, *bkdC*, *fadE2*, *fadE13*, *accD2* (implicated in the catabolism of branched-chain keto and amino acids); Proteases and peptidases: *pepD, pepR, htrA and clpC2; arcA* (arginine deiminase); *gcvB* (glycine dehydrogenase), which are involved in degradation of arginine and glycine, respectively; *hsaG* (involved in the degradation of aromatic compounds); *nuoA-N*; *sigA, sigB, sigE, sigF, sigG, sigH, sigI, sigL*, and *sigM*	Salina et al., 2014, 2019

**TABLE 2:** *Mycobacterium tuberculosis* proteins upregulated in *in vitro* models of dormancy.

Model	Method for achieving dormancy	Selected genes upregulated under hypoxia	References
Hypoxia	*M. tuberculosis* was inoculated into BACTEC vials, and incubated at 37 °C in a BACTEC460 apparatus. After 8–10 days half of the cultures were shifted to anaerobic growth conditions (85 % N_2_, 10 % H_2_, 5 % CO_2_). After 22–26 days the cultures were harvested and protein extracts were prepared.	Rv2005c [similar to universal stress proteins (USPs)], Gro-EL2; elongation factor Tu (Rv0685); β-ketoacyl-ACP, succinyl-CoA : 3-oxoacid-CoA transferase; cyclopropane mycolic acid synthase 2, thioredoxin reductase, L-alanine dehydrogenase (Ald) (Rv2780), Rv2629, Rv2185c, Rv0560c (probable SAM utilizing methyltransferase) and Rv3866	Starck et al., 2004
Hypoxia	Cultures were grown in Dubos Tween-albumin broth in a fermentor to mimic Wayne’s model Dissolved oxygen (DO) was monitored. Cells were harvested when DO indicated achievement of log phase, NRP-1 or NRP-2	13 proteins of the DosR regulon, PckA (phosphoenolpyruvate carboxykinase), trehalose biosynthesis related proteins (GlgX, GlgY, GlgZ, and OtsB); Rv0082, 0571c, 0846c, 1047, 1326c, 1894c, 1998c, 3503c, and 3515c	Choa et al., 2006
Hypoxia	Wayne model	DosR regulon (HspX, TB31.7); Ald, SigB, SigE, ClgR, PrpC, PrpD; several proteins involved in lipid metabolism (FadE5, DesA1/2, Tgs1/4, and Icl); copper stress-related enzymes MymT (copper toxicity protection) and CsoR (copper-sensitive operon repressor), PfkB (phosphofructokinase B)	Schubert et al., 2015
Hypoxia	Wayne model	HspX (NRP1 and NRP2), Ald (NRP2), Rv2005c; DosR (NRP1), BfrB (NRP1), pyruvate dehydrogenase (NRP1), citrate synthase (NRP1), Rv1623, a subunit of cytochrome D terminal oxidase complex (NRP2), QcrA (Rv2195) (NRP1), sulfate transporters CysA2 and CysA3, ClpX (Rv2457c) (NRP1 and NRP2), deamidase of Pup (Dop and Rv2112c) (NRP2), FabG, KasB and FbpA. Transcriptional regulators: Rv0818, Rv0981, SigK, CspA, Rv2258c, PrrA, WhiA, and DosR were up-regulated during NRP1. MprA, SigK, Rv1019, HrcA, Crp, DosS, and DosR were up-regulated during NRP2	Gopinath et al., 2015
Nutrient starvation	Model of Betts et al. (2002)	Transcriptional repressors CmtR, Rv0144, Rv0158, Rv0328, Rv1219c, Rv1556, Rv3295, Rv3557c; the serine threonine kinase PknH; 11 members of the TA family (MazF6, ParE2, RelE2,VapB32, VapC13, VapC19, VapC22, VapC39, VapC4, VapC41, VapC5); AtpA, C, D, and G which form the ATP synthase enzyme complex; putative iron(III)-siderophore substrates (FecB and FecB2); the molybdate transport system (ModA), and phosphate uptake (PstS1 and PstS2);HemC, CysG, HemZ, and Rv1314c, involved in porphyrin biosynthesis; several lipoproteins. Enoyl CoA hydratases (EchA1, EchA4, EchA5, EchA7,EchA8, EchA15, EchA16, EchA19), lipoproteins LpqK, LpqL, LpqM, SodC, PstS2, PstS1, LpqT, LpqW LpqX, LpqZ, LprA Possible lipoprotein LprA, OppA; stringent response regulator	Albrethsen et al, 2013
Potassium depletion	Described in Table 1	Ald, Wag31, RibA2, PpiA, FabG4, FixA and EchA6	Salina et al., 2014
Hypoxia in the presence of cholesterol		FadA5 (NRP1), TB31.7 (NRP1 And NRP2). HspX (NRP1), TB31.7 (NRP2); bacterioferritin protein BfrB (NRP2), FadA5 (NRP1), FixB, (NRP2)	Garcia-Morales et al., 2017

Intragranulomatous lesions are believed to harbor dormant *M. tuberculosis* populations. The granulomata from different TB pathologies provide distinct microenvironments for *M. tuberculosis*. The lesions associated with active TB (ATB) are less hypoxic than those associated with LTBI. Hypoxia within the LTBI lesions is therefore a trigger for the bacterium to enter dormancy. Hudock et al. (2017) have analyzed gene expression in defined microanatomic compartments of the lungs of non-human primates with active or latent TB. A core group of 633 genes were identified associated with both ATB and LTBI, suggesting that these are required for *M. tuberculosis* survival. The *dosR* regulon was expressed at the lowest level in the least hypoxic lesions, and at the highest level in the most hypoxic lesions (Hudock et al., 2017).

### Other Two-Component Systems

*mprA* and *regX3* were identified as components of a core temporal regulatory response during 0.25–8 h of vitamin C treatment (Nandi et al., 2019). *phoP* was induced at an intermediate temporal window during vitamin C treatment. Potassium depletion was also associated with the induction of the *mprA*B TCS. In addition, in this model, the sensor kinase *kdpD* of the TCS *kdpDE* (linked to potassium transport) and the response regulator *prrA* of the TCS *prrAB* (required for macrophage infection), were induced. *kdpDE* was also induced by nutrient starvation (Betts et al., 2002). Among the less well understood TCSs, *narL* and *tcrA* were upregulated following vitamin C treatment of *M. tuberculosis*. Proteomics confirmed the induction of DosR/DosS, MprA, and PrrA under hypoxia (Gopinath et al., 2015).

### Sigma Factors

*In vitro* models of dormancy (most notably hypoxia, vitamin C exposure, potassium depletion and nutrient starvation) have reported the induction of several of the transcripts of extracytoplasmic function (ECF) sigma factors. Almost all the *in vitro* models have confirmed that *sigE*, *sigH*, and *sigB* are induced during dormancy (Betts et al., 2002; Voskuil et al., 2004; Salina et al., 2014, 2019; Iona et al., 2016; Nandi et al., 2019). In addition, the upregulation of *sigF* and *sigD* and several other sigma factors, has also been reported in multiple studies. These are summarized in Table 1. *sigB* was induced at 10 days of hypoxia and remained induced upto 30 days. *sigE* and *sigC* were induced early during hypoxia and fell off at day 10 and day 12, respectively. *sigH* was induced throughout hypoxia (Voskuil et al., 2004). *In vitro* studies have also shown that *sigE, sigB*, and *sigH* respond to surface stress and oxidative stress, respectively, and that the transcription of *sigB* under surface stress is dependent on *sigE* (Manganelli et al., 2001, 2002). *sigB*, *sigH*, *sigF*, s*igM*, and *sigE* were enriched during vitamin C treatment of *M. tuberculosis* (Nandi et al., 2019). In the potassium depletion model of dormancy, genes *sigA, sigB, sigE, sigF, sigG, sigH, sigI, sigL*, and *sigM* were induced; *sigD, sigJ*, and *sigK* were repressed (Salina et al., 2014). Proteomic studies also confirmed the induction of SigK during NRP1 (Gopinath et al., 2015) (Table 2).

Intragranulomatous lesions from different TB pathologies in non-human primates [i.e., active TB (ATB) or latent TB (LTBI)] (Hudock et al., 2017) showed the expression of *sigB*, *sigD*, *sigI*, *sigJ*, and *sigF* in all the lesions. LTBI was associated with *sigL* and *sigM* (caseum), as well as *sigK* and *sigG* (granuloma) expression. These results corroborated at least in part, some of the observations made from the *in vitro* models of dormancy.

### Transcriptional Regulators

A lipid-rich microenvironment is the hallmark of the granuloma. *In vitro* models have therefore tested the response of *M. tuberculosis* to growth in lipid-rich medium. During growth in the presence of lipids, ClgR appeared to be the predominantly upregulated transcriptional regulator under NRP1 (Del Portillo et al., 2019). On the other hand, Rv0081 was the more predominantly expressed transcriptional regulator during hypoxia in dextrose medium, although it is also induced in fatty acid-grown cells at stationary phase (Rodríguez et al., 2014). A core of 185 genes is upregulated during growth in the presence of long chain fatty acids (Aguilar-Ayala et al., 2017). Among these are the transcriptional regulators Rv3160c and Rv0678. Rv3160c is a TetR-like transcriptional repressor that regulates expression of the putative oxygenase Rv3161c (Tükenmez et al., 2021). Rv0678 encodes the MmpR repressor protein, responsible for regulating the transcription of MmpL5 and MmpS5 protein which together make up the MmpL5-MmpS5 efflux pump associated with cross-resistance between clofazimine and bedaquiline (Hartkoorn et al., 2014).

The WhiB family of proteins of *M. tuberculosis* represent a group of iron-sulfur containing redox sensing transcriptional regulators which respond to stress and maintain redox homeostasis (Singh et al., 2009; Larsson et al., 2012). Among these, *whiB1* was upregulated during vitamin C treatment (Nandi et al., 2019) and potassium depletion (Salina et al., 2014); *whiB2* was upregulated during nutrient starvation (Betts et al., 2002); *whiB3* was upregulated in fatty acid grown cells at stationary phase (Rodríguez et al., 2014), and during treatment of *M. tuberculosis* with vitamin C (Nandi et al., 2019) and *whiB6* was upregulated during potassium depletion (Salina et al., 2014, 2019).

Analysis of the transcriptional regulatory network that underlies the response to vitamin C, showed an early response (upto 1 h), an intermediate response (between 2 and 8 h) and a late response (24 h). The early response regulators induced included *Rv0348*, *hrcA*, and *Rv0845*. The late response included the regulators *lsr2*, *Rv0081*, *Rv0678*, *trcR*, and *Rv0047*. Nutrient starvation resulted in the upregulation of *Rv2034*, *Rv1152*, *Rv3291c* (Betts et al., 2002). *kstR*, a transcriptional repressor controlling cholesterol catabolism was induced in non-culturable bacilli resulting from potassium depletion (Salina et al., 2014).

Proteomic analyses confirmed the upregulation of the stringent response regulator RelA (Rv2583c), and Rv1019 (a tetR family transcriptional regulator), during hypoxia (Gopinath et al., 2015).

Peterson et al. (2020) reported a transcriptional program that coordinates sequential state transitions to drive *M. tuberculosis* in and out of hypoxia-induced dormancy. This model employed an accurate technique for generating a defined oxygen gradient, where dissolved oxygen levels can be monitored. Non-overlapping sets of differentially expressed genes (DEGs) were associated with each of the following states: normoxia (81 genes), depletion (446 genes), early hypoxia (328 genes), mid hypoxia (320 genes), late hypoxia (978 genes), and resuscitation (429 genes). The hypoxia dataset was linked to a transcription factor (TF) gene network derived from chromatin immunoprecipitation sequencing (ChIP-seq) (Minch et al., 2015) in order to understand the transcriptional regulation of the transition between the aforesaid states. Network motifs such as feed forward loops (FFLs) and TFs which figure as central players in these FFLs such as Rv0081, were identified. The *Rv0081-Rv0324* FFL was predicted to be responsible for controlling the late hypoxia genes. The regulatory activity of Rv0081 appeared to be oxygen-dependent, and the oscillations of state observed as oxygen dropped below 3%, could be due to Rv0081-directed incoherent FFLs (I-FFLs). The I-FFL controlled by Rv0081 probably regulates the transition to late hypoxia and imparts robustness into the hypoxic response.

### Genes/Proteins Linked to Replication, Transcription, and Translation

Ribosomal protein encoding genes *rpsR2*, *rpsN2* and *rpmG1* were induced early during hypoxia, but repressed at later stages (after day 20) (Voskuil et al., 2004). In line with reduced requirements for mRNA and protein synthesis during dormancy, the 30s and 50s ribosomal protein genes (*rpsJ-rpsQ*, *rplN-rpsN*, and *rpsH-rpmD*) were downregulated in the vitamin C-induced model of dormancy (Nandi et al., 2019). *rrnAP1* and *rrnAPCL1* involved in ribosomal RNA synthesis were downregulated during dormancy (Iona et al., 2016). Genes linked to chromosome partitioning (*parA* and *parB*), 15 genes in the cluster Rv0700 to Rv0723, involved in ribosomal protein synthesis, *rpoA* and *rpoC* (subunits of RNA polymerase), aminoacyl tRNA synthases (*gltS* and *trpS*) were all downregulated during nutrient starvation (Betts et al., 2002). The vitamin C induced dormancy model showed the downregulation of GreA transcription elongation factor, 30s and 50s ribosomal proteins (*rpsL, rpsL, rpsJ, rplC, rpsQ, rplN, rplX, rpmE*, and *rpmB2*), and *dnaA* and *dnaB* (which interact with the origin of replication) (Nandi et al., 2019). Rodríguez et al. (2014) reported that bacteria grown in the presence of even chain fatty acids (F) till stationary (S) phase (FS phase) show a remarkable overexpression of tRNAs, a probable reflection of low translation activity. tRNA-Lys, tRNA-Ala, and tRNA-Arg neutralize the negative charge on the polar head groups of phosphatidylglycerol. These tRNAs were overexpressed in FS compared to DS. This could possibly be linked to changes in membrane permeability and decreased susceptibility to antibacterial cationic drugs.

### Electron Transfer Processes and Aerobic Respiration

Cytochrome *bd* oxidase (encoded by *cydABCD*), and cytochrome *bc1*-*aa*_3_ regulate respiratory functions in *M. tuberculosis*. The cytochrome bd oxidase encoded by the *cydABDC* cluster consists of *cydAB* (encoding cytochrome bd oxidase) and *cydCD* (encoding the ABC transporter). Voskuil et al. (2004) have shown that the *cydABCD* cluster is induced early during hypoxia, consistent with a role of cytochrome bd oxidase as an alternative terminal oxidase for the aerobic respiratory chain that functions under low oxygen levels. On the other hand *cydAB* as well as cytochrome c reductase (*qcrA* and *qcrC*), and cytochrome c oxidase (*ctaC* and *ctaE*) were repressed under potassium depletion-induced dormancy. NADH dehydrogenase genes (*nuoA-N*) were repressed in non-culturable bacteria under potassium depletion (Salina et al., 2014) and under nutrient starvation (Betts et al., 2002), whereas the uncoupled non-proton pumping NADH dehydrogenase (*ndh*) was induced in non-culturable bacteria (Salina et al., 2014). This suggested that during potassium depletion induced dormancy, the bacilli switch from using proton motive force generated by respiration to using NADH and alternative electron acceptors. Genes of the NADH dehydrogenase complex and the ATP synthase were also downregulated when bacteria were treated with vitamin C (*nuoH-nuoN*, *nuoC-G*, and *atpC-H*) (Nandi et al., 2019) or subjected to nutrient starvation (*nuoA-M* and *atpA-H*) (Betts et al., 2002).

Proteomic studies further confirmed the decreased abundance of proteins involved in aerobic respiration (NuoE, NuoF, and NuoG), and quinolate synthase (NadA) (involved in the biosynthesis of NAD) during nutrient starvation (Albrethsen et al, 2013). Intriguingly, Gopinath et al. (2015) could not demonstrate diminished levels of the subunits of ATP synthase during hypoxia. FixB, an electron acceptor flavoprotein of dehydrogenases at complex II of the cell respiratory chain was overexpressed in NRP2 when cells were grown in the presence of cholesterol as carbon source (Garcia-Morales et al., 2017), most likely to maintain redox balance inside the cell.

### Genes and Proteins Linked to Metal Ion Storage and Acquisition, and Transport of Inorganic Ions

The bacterium needs to increase iron stores during dormancy. Expectedly, the iron storage protein bacterioferritin (*bfrB*) (Voskuil et al., 2004) and mycobactin synthesizing gene cluster (*Rv2377c to Rv2386c, mbtA-I*) were induced during hypoxia (Bacon et al., 2004; Voskuil et al., 2004). Muttucumaru et al. (2004) confirmed the upregulation of the mycobactin biosynthesizing operon, and ferredoxin A (*fdxA*) during hypoxia. Copper is required by *M. tuberculosis* for survival, but copper overload can be toxic (Rowland and Niederweis, 2012). *ctpV* (an efflux pump) and *ctpG* (a P-type ATPase), which prevent copper toxicity, were upregulated during vitamin C treatment (Taneja et al., 2010; Sikri et al., 2015). *ctpF*, a cation transport ATPase was upregulated during hypoxia (Voskuil et al., 2004). The molybdate transport system (*modA*) and phosphate uptake system (*pstS1* and *psS2*) were increased during nutrient starvation suggesting increased transport of iron, molybdate and phosphate under these conditions. However, following vitamin C treatment, the phosphate-specific transporter operon *pstB-pstC1-pstA2*, was downregulated. The sulfate transporters *csyT–cysW–cysA* are induced under nutrient starvation (Betts et al., 2002).

Schubert et al. (2015) have shown through proteomic analyses, that the copper stress-related enzymes MymT (copper toxicity protection) and CsoR (copper-sensitive operon repressor) were induced during hypoxia. Gopinath et al. (2015) have shown that the sulfate transporters CysA2 and CysA3 were upregulated in both dormancy and reactivation. Sulfate is required for the production of methionine and cysteine. Therefore it is possible that sulfate transport regulates reductive stress. Putative iron (III)-siderophore substrates (FecB and FecB2) the molybdate transport system (ModA), and phosphate uptake (PstS1 and PstS2), suggesting increased transport of iron, molybdate, and phosphate during starvation (Albrethsen et al, 2013). Gopinath et al. (2015) have reported the upregulation of the iron storage protein BfrB during hypoxia.

### Central Carbon Metabolism

Several independent studies have confirmed that central carbon metabolism slows down during dormancy or a shift of the bacterium to a non-culturable state. When fatty acids or cholesterol is the sole carbon source for *M. tuberculosis*, acetyl-CoA and propionyl-CoA must be metabolized via the glyoxylate and methylcitrate cycle, respectively. The glyoxylate cycle requires the enzyme isocitrate lyase 1 (*icl1*). Growth in the presence of long chain fatty acids has shown a shift in metabolism to increased expression of *icl1* (Rodríguez et al., 2014). *pckA* (required for growth on fatty acids) (Marrero et al., 2010) was also upregulated. Vitamin C treatment was associated with overexpression of *icl1* (Nandi et al., 2019). Genes linked to glycolysis and the tricarboxylic acid (TCA) cycle (*fum*, *acn*, and *icd1*) were downregulated under nutrient starvation (Betts et al., 2002). The genes *pdhABC* encoding subunits of the pyruvate dehydrogenase enzyme complex, the fumarate reductase complex (*frdABCD*),were upregulated under nutrient starvation.

Salina et al. (2014, 2019) have analyzed transcriptional signatures generated for bacilli cultured in potassium-depleted medium to show that genes linked to glycolysis and gluconeogenesis are repressed in the non-culturable bacilli, with *pgi*, *fba*, *tpi*, *gap*, *pgk*, *pgmA*, *eno*, *pykA*, *aceE*, and *lpdC* downregulated compared to bacteria grown in potassium-sufficient media (Salina et al., 2014, 2019). Four genes implicated in the pentose phosphate shunt were also repressed (*fgd1*, *zwf2*, *tkt*, and *tal*). The tricarboxylic acid cycle (TCA) genes *citA, acn, icd1, icd2, korA, korB, sucC, sucD, shdA, shdB, shdD, Rv0248c* (probable succinate dehydrogenase), *fumC* and *gltA2* were downregulated. *icl1* was induced as was *mutA* of the methylmalonyl pathway. Bacon et al. (2004) have reported the induction of *prpD*, which is involved in the methylcitrate cycle, under hypoxic conditions. Eoh and Rhee (2013) have suggested that Icl-mediated synthesis of succinate may afford *M. tuberculosis* an efficient means of entry into and exit from hypoxia-induced dormancy.

Proteome analysis of cells under hypoxia showed the upregulation of pyruvate dehydrogenase and citrate synthase at NRP1 (Gopinath et al., 2015). However, increased expression of Icl was not observed. Choa et al. (2006) have reported the upregulation of PckA (phosphoenolpyruvate carboxykinase) in NRP-1 when mycobacteria are grown on fatty acid substrates, suggesting a shift to gluconeogenesis from lipid precursors.

### Lipid Metabolizing Genes/Proteins

During entry into dormancy, mycobacteria maintain an equilibrium between fatty acid biosynthesis and degradation, while fatty acid biosynthesis surges during reactivation. Mycolic acid-biosynthesizing genes were generally observed to be downregulated in different models of dormancy. *accA2* and *accD2* (Barry et al., 2007), were downregulated during vitamin C treatment (Taneja et al., 2010) and hypoxia (Bacon et al., 2004). *mmA3* (a methyltransferase that generates mycolates), (Behr et al., 2000), was downregulated during vitamin C treatment (Taneja et al., 2010). *tgs1* (involved in triglyceride synthesis), *fadE5*, *fadE13*, and *fadD19* (involved in fatty acid degradation) (Muñoz-Elías and McKinney, 2005); *fadE6*, *fadE28*, and *fadE32* (putative acyl-CoA dehydrogenases), *scoA and scoB* (involved in the utilization of ketones) were upregulated during vitamin C treatment. *desA3* a desaturase involved in oleic acid synthesis (Walker et al., 1970), was also downregulated. While several genes of fatty acid degradation were upregulated during dormancy, selected genes (*fadD5*, *fadD10*, *fadD22*, *fadD29*, and *fadD30*) were dowregulated in the vitamin C model. During nutrient starvation, *desA3* and *desA2*, the polyketide synthase type I system, *ppsABCDE*, the type II polyketide synthase, *mas*, which are involved in the synthesis and transport of phthiocerol dimycocerosates (PDIMs), and the mycolyl transferases *fbpA* and *fbpB*, were downregulated (Betts et al., 2002). Genes implicated in the catabolism of branched-chain keto acids (*bkdA, bkdB*, *bkdC, fadE2, fadE13*, and *accD2*) were upregulated. Fatty acid and mycolic acid biosynthetic pathway genes (*fabG1* and *inhA* from FASII, *fas, accD4, mmaA2, mmaa4, cmaA2, umaA*, and *Rv2509*) were repressed during potassium depletion induced dormancy (Salina et al., 2014, 2019). *fadD26* a fatty acyl AMP ligase involved in the biosynthesis of PDIMs (Siméone et al., 2010) was upregulated in NRP1 and NRP2 (Muttucumaru et al., 2004).

Proteomics revealed the upregulation of FadA5 during hypoxia in the presence of cholesterol (Garcia-Morales et al., 2017). In the potassium-depleted non-culturable state (Salina et al., 2014), KasB (involved in fatty acid biosynthesis) was decreased compared to exponentially growing cells. FadE5, DesA1/2 and Tgs1/4 were induced during hypoxia-induced dormancy (Schubert et al., 2015). Starck et al. (2004) grew bacteria aerobically for 8–10 days and then shifted to anaerobic growth conditions and analyzed the cytosolic proteome of *M. tuberculosis*. The L-alanine dehydrogenase (Ald, Rv2780) (which converts pyruvate to alanine, and glyoxylate to glycine concurrent with the oxidation of NADH to NAD) (Giffin et al., 2016) was upregulated under hypoxic conditions. Succinyl-CoA : 3-oxoacid-CoA transferase (ScoB, Rv2503c), which catalyses the reversible conversion of succinyl-CoA to succinate, was upregulated, as was β-ketoacyl-ACP synthase (KasB and Rv2246) which is involved in the synthesis of mycolic acids (Schaeffer et al., 2001). A total of about 50 proteins were unique under anaerobic conditions and low ATP levels. A selected list of proteins upregulated during dormancy is given in Table 2. Gopinath et al. (2015) observed that six proteins linked to fatty acid degradation were upregulated at NRP1 and NRP2 and confirmed that FabG, KasB and FbpA were upregulated during hypoxia. EchA6 and FabG4, involved in fatty acid oxidation and fatty acid biosynthesis, respectively were induced during potassium depletion.

### Trehalose

Trehalose can serve as a carbon source, as a storage carbohydrate, and as an osmoprotectant in the non-replicating bacteria. It also regulates the host immune response. The OtsAB pathway is the dominant pathway for trehalose synthesis and is required for *M. tuberculosis* grown in culture and for virulence in a mouse model. *otsB*, a trehalose 6 phosphate phosphatase was induced during dormancy (Voskuil et al., 2004). Choa et al. (2006) have shown that four of the trehalose biosynthesis related proteins (GlgX, GlgY, GlgZ, and OtsB) are upregulated in NRP-2.

### ESX Secretion Systems

Albrethsen et al (2013) have analyzed the secreted proteome of *M. tuberculosis* under nutrient starvation. The Esx secretion system members, EsxA, EsxB, EsxJ/EsxK, EsxL, and EsxO, all showed decreased abundance. Iona et al. (2016) have reported downregulation of EsxA and EsxB during hypoxia-induced dormancy.

### Proteases and Peptidases

The Clp protease is a key regulator of the response to stress. Proteomic analyses have shown that ClpX (Rv2457c) is upregulated during NRP1 and NRP2, whereas ClpP1 and ClpP2 are present at normal levels (Albrethsen et al, 2013). Mycobacteria possess a prokaryotic ubiquitin-like protein (Pup) proteasome pathway. The enzyme deamidase of Pup (Dop, Rv2112c) deamidates the C-terminal glutamine of Pup to form glutamate, thereby activating Pup. Dop was dramatically upregulated during NRP2, and its level returned to normal as the number of proteins began to increase during re-aeration. During potassium depletion, *pepD* (encoding an HtrA-like serine protease), *htrA* and *clpC2* were upregulated (Salina et al., 2019).

### Chaperones

Chaperone-encoding genes such as *hspX*, *dnaK*, *dnaJ1*, *grpE*, *clpB*, *hsp* (or *acr2*), and *htpX* were upregulated after Vitamin C treatment (Taneja et al., 2010; Sikri et al., 2015; Nandi et al., 2019). Also induced was *trxB1* (encoding thioredoxin).

Proteome analyses showed the upregulation of the universal stress protein Rv2005c and HspX (Rv2031c) during dormancy (Gopinath et al., 2015; Garcia-Morales et al., 2017). HspX also exhibited high expression in LTBI and ATB caseum-derived samples obtained from the lungs of non-human primates (Hudock et al., 2017).

### Toxin-Antitoxin Systems

Type II toxin–antitoxin (TA) systems are widely spread among bacteria and archaea. Type II TA systems are involved in persistence regulation, antibiotic tolerance, stress adaptation and virulence (Maisonneuve et al., 2011; Leung and Lévesque, 2012; De la Cruz et al., 2013). Aguilar-Ayala et al. (2017) reported the overexpression of toxin/antitoxins *vapB9/vapC9* and *vapB22/vapC22* during hypoxia in the presence of long chain fatty acids. Stationary phase in the presence of long chain fatty acids and cholesterol was associated with overexpression of *vapC1*, *vapC22*, *vapB1*, *vapB10*, *vapB46*, and *vapB48*. Bacon et al. (2004) reported the hypoxia-induced induction of *higBA3* (Rv3182–Rv3183) (Zaychikova et al., 2015). *vapB21*, an antitoxin of the TA system, was induced approximately 100-fold in the granulomatous lesions derived from non-human primates with active TB (Hudock et al., 2017). Proteomic analysis by Del Portillo et al. (2019) showed the induction of the TA module proteins VapB10, VapC37, and VapC20 in dextrose-grown cells under hypoxia.

### Non-coding RNAs

Rodríguez et al. (2014) suggest that adaptation of *M. tuberculosis* during growth in the presence long chain fatty acids as sole carbon sources, leads to a slow growth and drug-tolerant phenotype, characteristic of the dormant state. Gene expression in *M. tuberculosis* grown in a fatty acid (F) environment was compared with growth on dextrose containing (D) medium, using strand-specific RNA sequencing (Wang et al., 2009). Genes with higher expression during growth on LC-FAs were compared with the highest scoring dormancy-associated genes obtained in the meta-analysis of published microarray data by Murphy and Brown (2007), and a high degree of overlap was observed. Mycobacterial small RNAs modulate the response of mycobacteria to the environment (Arnvig and Young, 2009). Most of the more than 200 sRNAs of mycobacteria are ncRNAs (Haning et al., 2014). The most highly expressed ncRNA, MTS2823 (ncRv13661A) is upregulated in fatty acid grown cells. Del Portillo et al. (2019) established hypoxic cultures of *M. tuberculosis* after exponential phase growth in the presence of long chain fatty acids or dextrose. Two non-coding RNAs (ncRNAs), MTS1338 and MTS0194 were upregulated in the fatty acid grown cells only. MTS2823 and the stable 10S RNA were expressed in high levels in NRP2 in both carbon sources. Aguilar-Ayala et al. (2017) also observed that non-coding RNA MTS2823 is upregulated during growth in the presence of fatty acids.

## Genes and Proteins Differentially Expressed During Reactivation From Dormancy

### Transcriptional Regulators

The DosRST regulon and the MprAB regulon genes which play important roles in the response to stress (He et al., 2006; Pang et al., 2007) were downregulated during reactivation from hypoxia, including Rv0081 and its subnetwork. Among the sigma factors, the *sigH* and *sigE* network genes were downregulated, including ClgR and its targets Rv2743c and Rv2744c. This was in harmony with the role of the ClgR regulon in maintaining cell envelope functions under stress (Estorninho et al., 2010; Datta et al., 2015). These findings were in contrast to those of Sherrid et al. (2010), who subjected bacteria to hypoxic conditions for a shorter period of 7 days. In their study, a number of genes of the MprA subnetwork were upregulated.

The TetR-like transcriptional repressor KstR that represses the expression of a cluster of mycobacterial genes involved in cholesterol catabolism (Kendall et al., 2007; Van der Geize et al., 2007), was downregulated, consistent with heightened cholesterol catabolism under stress such as hypoxia.

The SWATH analysis (Schubert et al., 2015) identified a cluster of proteins that were transiently upregulated within 6 h of re-aeration, including the sigma factors SigE and SigB and the transcriptional regulator ClgR, suggesting their involvement early in resuscitation, but not at the later stages.

During reactivation from potassium depletion, the transcriptional regulators WhiB6 [which regulates cell division (Chen et al., 2016)] and TetR family regulators Rv3830c and Rv3160c which regulate multidrug efflux pumps, as well as the response to osmotic stress and toxic chemicals, were upregulated.

### Energy Metabolism and Electron Transport Chain

Du et al. (2016) analyzed reactivation of *M. tuberculosis* after 25 days in the Wayne model of hypoxia. Early during reactivation, there is a dramatic increase in cellular ATP levels. In preparation for replication, *M. tuberculosis* upregulates pathways involved in ribosome biosynthesis and amino acid biosynthesis. Genes for TCA cycle and oxidative phosphorylation enzymes such as NADH:quinine oxidoreductase and F-type ATPase were upregulated. Sherrid et al. (2010) have shown that among genes of the MprA subnetwork, those encoding components of the NADH dehydrogenase complex (*nuo*) and ATP synthase (*atp*) were upregulated, consistent with increase in aerobic activity and ATP synthesis during reactivation.

The SWATH proteome analysis (Schubert et al., 2015), showed that the FoF1 ATP synthase levels did not change significantly over the time course of the experiment, underlining the requirement of this enzyme in maintaining ATP homeostasis in dormant *M. tuberculosis* (Gengenbacher et al., 2010; Leistikow et al., 2010). In contrast, components of the electron transport chain and energy generating machinery, changed in response to hypoxia, suggesting a reorganization of energy metabolism.

### Central Carbon Metabolism

Genes involved in respiration, TCA cycle activity and translation were activated after 4 days of resuscitation from potassium-depleted to potassium-sufficient conditions. The SWATH analysis (Schubert et al., 2015) identified a cluster of proteins that were transiently upregulated within 6 h of re-aeration, but returned to pre-aeration levels within 2 days. This included PrpC and PrpD, two enzymes of the methylcitrate cycle.

### Lipid Biosynthesis

There was upregulation of key enzymes involved in the biosynthesis of mycolic acids and sulfolipids during reaeration after hypoxia. The gene *fabJ* which links the FAS-I and FAS-II systems (Choi et al., 2000), was upregulated in the initial phase of reaeration. The genes *fabG1* and *inhA* were also induced, indicative of a shift toward mycolic acid biosynthesis. The genes *mas*, *faD26*, and *ppsA-E*, involved in the synthesis and translocation of PDIM, were upregulated, in line with the observations of Sherrid et al. (2010). Several *pks* genes (*pks1*, *psk4*, *pks7*, *pks8*, and *pks15*) encoding polyketide synthases that participate in the synthesis of complex lipids including sulfolipids were induced. Taken together, the observations pointed to enhanced synthesis of major cell wall lipids in *M. tuberculosis* during the emergence from dormancy and preparation for cell division. Lipid catabolism is associated with entry of *M. tuberculosis* to the persistent state. Cholesterol uptake and utilization are required for *M. tuberculosis* survival during persistence (Pandey and Sassetti, 2008). Expectedly, genes associated with fatty acid beta-oxidation and degradation pathways were downregulated in the reaeration phase. Also downregulated were the Mce transport systems which encode ABC transporters involved in transport of diverse lipids across the cell wall (Casali and Riley, 2007; Pandey and Sassetti, 2008). Genes involved in glyoxylate and dicarboxylate metabolism, a canonical pathway for lipid utilization, were downregulated.

Proteome analysis during a shift from hypoxia to reaeration showed that four proteins involved in mycolic acid biosynthesis were upregulated at day 6 of reaeration, whereas cyclopropane mycolic acid synthase 2 (CmaA2) was downregulated (Gopinath et al., 2015). At day 24 of reaeration, 20 proteins were at normal levels and three (KasA, BacA, and FbpA) were upregulated.

### Cell Wall Synthesis, Division, DNA Replication, and Repair

Iona et al. (2016) have shown that when cells subjected to a 25 days period of dormancy were reactivated, *ftsZ* and *dnaA* associated with cell division and replication, were upregulated. The resuscitation promoting factors (Rpfs), *rpfB*, *rpfC* went up early, followed by the other *rpfs*. Shift from potassium depletion to potassium sufficient conditions showed that most of the *rpf* encoding genes were activated only after the onset of cell division. Only *rpfE* was upregulated at day 7, coinciding with the onset of multiplication. *rpfB* was upregulated after 8 days of resuscitation (Salina et al., 2014).

At least seven proteins involved in DNA replication and repair, (single-stranded DNA binding protein (SSB), Hns, FtsE, ParB, DNA polymerase I, PolA, DNA topoisomerase I TopA omega, and DNA polymerase III beta chain DnaN) were present at normal levels at day 6 of reaeration (Gopinath et al., 2015). At day 24, a further set of proteins appeared, including the NrdEF system which encodes an enzyme that catalyzes the formation of deoxyribonucleotides from ribonucleotides. Rv2817c (putative Cas1, a CRISPR-associated endonuclease) was detected in the early stages of reactivation, suggesting a possible role of genome editing during reactivation of *M. tuberculosis* from dormancy.

### ESX Secretion Systems

Peterson et al. (2020) have shown that a shift from late hypoxia into resuscitation was characterized by expression of the *ESX-5* export system (Gey van Pittius et al., 2001).

### Toxin–Antitoxins

A few toxins and antitoxins also showed expression specific to the reactivation [Rv0299 and RelE, in the early period of reactivation and Rv0298 in the later phase (Gopinath et al., 2015)].

### Chaperones

The universal stress protein Rv2005c was upregulated about 40-fold immediately upon reaeration, but decreased to 17-fold at R24. HspX (Rv2031c) was decreased 175-fold during R6 and 64-fold during R24 (Gopinath et al., 2015).

### Proteases, Transposases, and Insertion Sequences

Proteases, transposases and insertion sequences were also expressed during resuscitation, prompting the speculation that there is genome reorganization to facilitate the chances of the bacterium to survive and to transmit to a new host. The endopeptidase *clpB* which is required for recovery from the stationary phase or antibiotic exposure (Vaubourgeix et al., 2015) was upregulated during resuscitation from potassium depletion (Salina et al., 2014).

## Conclusion

*M. tuberculosis* remains in a dormant state, in latently infected individuals, with reactivation occurring when the immune system weakens. Targeting the dormant bacterium or triggering reactivation, represent important strategies for combating the disease. Omic approaches have provided valuable insights into how the bacterium remodels its transcriptome and its proteome during dormancy and reactivation. A feature of most of the models of dormancy was the upregulation of *dosR* and DosR regulon genes. A summary of selected genes or processes that are differentially regulated during dormancy is provided in Table 3. Besides *dosR*, the implications of upregulation of other components of TCSs during various time windows of dormancy, such as *mprA*, *regX3*, *prrA*, *kdpDE*, and *phoP*, remain poorly understood till date. Upregulation of the *narX-narK2* operon, suggests that nitrate transport into the cell occurs during dormancy. The *Rv0079* to *Rv0087* cluster, featuring the transcriptional regulator Rv0081 which likely directs FFLs which are crucial to the adaptation to hypoxia, the chaperones *acr* or *hspX*, *acr2*, *tgs1*, the regulator *clgR*, cation transporting ATPases, thioredoxin (*trxB1*), genes involved in fatty acid degradation, sigma factors such as SigE, SigB, and SigH, and genes/proteins of the mycobacterial Clp protease. Among the sigma factors that are induced under hypoxia, SigE regulates genes involved in fatty acid degradation and the glyoxylate cycle, such as *icl1* (Muñoz-Elías and McKinney, 2005). Genes regulated by *sigH* include *sigE*, *sigB*, DNA repair proteins, stress response proteins, and enzymes involved in thiol metabolism such as thioredoxin and thioredoxin reductase. A cyclopeptide lassomycin active against stationary phase bacteria, interacts with ClpC1 (Gavrish et al., 2014), making the Clp proteases, promising targets in dormant bacteria. Understanding the importance of the regulator ClgR during dormancy in a lipid-rich environment, is an important area for future investigation. ClgR is induced in response to both hypoxia and reaeration (McGillivray et al., 2015). It is a regulator of Clp proteases which are crucial for the degradation of misfolded proteins and therefore, for stress management. ClgR is likely a crucial regulator of the ability of *M. tuberculosis* to survive within the host under stress as well as to reactivate under suitable conditions.

**TABLE 3:** Summary of selected genes that are differentially regulated in various models of dormancy and resuscitation.

Dormancy		Resuscitation	
Up	Down	Up	Down
DosR regulon	Aerobic respiration	Genes linked to respiration, TCA cycle activity	DosR regulon
Chaperones such as *dnaK*, *clpB*, *hspX*, *dnAJ1*, *grpE*, and *acr2*	NADH dehydrogenase (*nuoA-N*)	Genes involved in DNA replication and repair	
Cation transporting ATPases	Genes linked to cell division and growth	Genes involved in synthesis of PDIMs, mycolic acids and sulfolipids	
Glyoxylate and methylcitrate cycle enzymes	Ribosomal proteins	Rpfs	
Alanine dehydrogenase	Genes involved in DNA replication	Virulence factors such as the toxin RelE	
Genes involved in fatty acid degradation and cholesterol catabolism	Universal stress protein Rv2005c		
Transcriptional regulators such as SigE and ClgR			
11 members of the TA family			
Bacterioferritin			

Genes linked to aerobic respiration and glycolysis, replication, transcription, translation and cell division, and NADH dehydrogenase (*nuo A-G*; *nuoH-N*) were repressed during dormancy. Degradation of even-chain-length or odd-chain-length fatty acids leads to the formation of acetyl-CoA, or propionyl-CoA, respectively. Degradation of the cholesterol side chain or ring structure also yields acetyl-CoA and propionyl-CoA (Ouellet et al., 2011). Acetyl-CoA and propionyl-CoA are metabolized via the glyoxylate and methylcitrate cycle, respectively (McKinney et al., 2000; Muñoz-Elías and McKinney, 2005; Muñoz-Elías et al., 2006), making these pathways important for bacterial survival *in vivo*. Rv0467 encodes *icl1* which acts as an isocitrate as well as methylisocitrate lyase, and is involved in both glyoxylate and methylcitrate cycles, (Gould et al., 2006). The *prpDC* (Rv1130–1131) operon encodes two enzymes, methylcitrate dehydratase and methylcitrate synthase, which are involved in the methylcitrate pathway. Mutations in *prpR*, a transcriptional activator of the *prpDC* operon, have been associated with drug tolerance in *M. tuberculosis* (Hicks et al., 2018). As expected *icl1* and *prpDC* were induced in dormancy.

Proteomic studies have corroborated several of the findings from transcriptomics under dormancy, while raising some questions. The enzyme, Ald is induced under hypoxia (Starck et al., 2004; Gopinath et al., 2015; Schubert et al., 2015) as well as potassium depletion (Salina et al., 2014). This enzyme is suggested to generate alanine for protein and peptidoglycan synthesis. Alanine synthesis is coupled to NADH oxidation (Hutter and Dick, 1998). It is possible that the induction of Ald activity supports the maintenance of the NAD pool when oxygen becomes limiting. Tgs1 (Schubert et al., 2015), PckA (phosphoenolpyruvate carboxykinase), and trehalose biosynthesis related genes/proteins (Voskuil et al., 2004; Choa et al., 2006) were induced under hypoxia. The bacterioferritin protein BfrB (involved in iron storage), was induced in NRP1 (Voskuil et al., 2004; Gopinath et al., 2015), and then declined. Garcia-Morales et al. (2017) reported its induction during hypoxia in cholesterol-containing medium. Whereas several models have shown that *prpDC*, *sigE*, *sigB*, and *clgR* are induced during dormancy (Table 1), Schubert et al. (2015) have reported their induction at the protein level during reactivation as well, suggesting probable roles of these molecules during both dormancy and resuscitation. This could be an interesting avenue for future investigations.

Genes/proteins involved in cell division, replication, ribosomal protein synthesis, transcription and translation were expectedly downregulated during dormancy and upregulated during various phases of reactivation.

The role of ncRNAs in dormancy and resuscitation, is another area which is largely unexplored. Several studies have reported the upregulation of the ncRNA MTS2823 in different models of dormancy (Rodríguez et al., 2014; Aguilar-Ayala et al., 2017; Del Portillo et al., 2019). Arnvig et al. (2011) have suggested that the *prpCD* operon is one of the major targets of MTS2823. Deregulation of the RNA polymerase complex occurs when it is released from sequestration by MTS2823, leading to overexpression of the PrpBCD system (Płociński et al., 2019). MTS2823 is linked to the slow-down of *M*. *tuberculosis* growth (Arnvig and Young, 2012). Taken together, these observations suggest that MTS2823 could play a role in dormancy by regulating propionate metabolism.

There have been fewer omic studies capturing the transcriptional and proteomic network of *M. tuberculosis* during reactivation, compared to studies in various models of dormancy. Genes involved in respiration, TCA cycle activity, DNA replication and repair, synthesis of PDIMs, mycolic acids and sulfolipids, were activated during resuscitation (Table 3). The five genes encoding Rpfs were activated at different stages of reactivation. The DosRST regulon was downregulated. Proteases, transposases and insertion sequences were also expressed, suggesting genome reorganization during reactivation.

**FIGURE 1:**
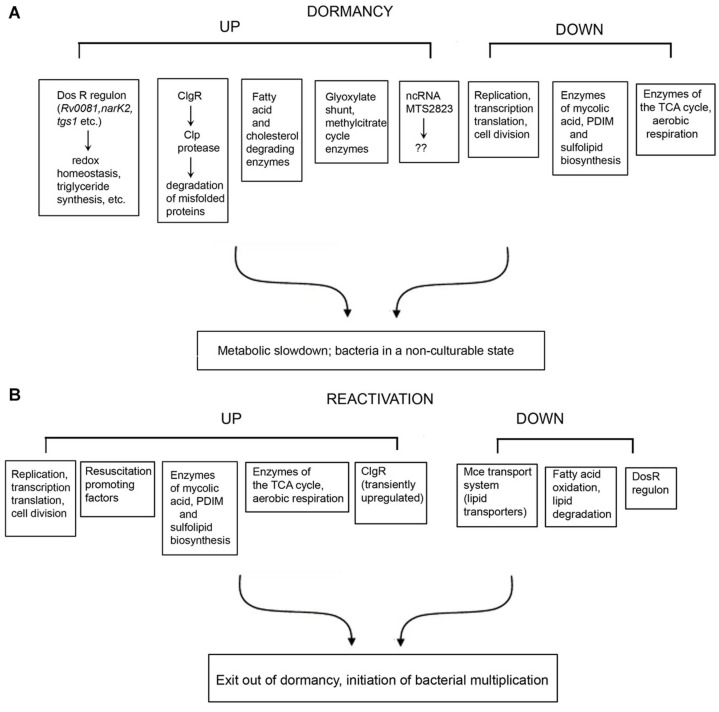
Summary of selected genes, proteins and pathways that are differentially regulated under dormancy **(A)** and reactivation **(B)** of *Mycobacterium tuberculosis*.

The genes and/or processes that are differentially regulated during dormancy and reactivation, are summarized in Figure 1. There are contradictions in the reports from different laboratories, and between transcriptomic and proteomic data. For example, Bacon et al. (2004) reported that *bfrB* is unchanged during hypoxia, whereas other studies have reported its induction under hypoxia (Voskuil et al., 2004; Gopinath et al., 2015). Contrary to other reports, Gopinath et al. (2015) failed to detect induction of Icl1 under hypoxia. While Voskuil et al. (2004) reported the induction of *nrdZ* (a ribonucleotide reductase class II enzyme that converts nucleoside triphosphates to deoxynucleoside triphosphates) during prolonged hypoxia, this could not be corroborated by Bacon et al. (2004). Voskuil et al. (2004) have reported that the four genes encoding the cytochrome bd oxidase were induced early during hypoxia whereas cytochrome bd oxidase was repressed in the potassium depletion model of dormancy (Salina et al., 2014, 2019). Most mycolic acid synthesizing/modifying genes were downregulated under dormancy (Sikri et al., 2015). However, Sikri et al. (2015) observed that *accA2* and *accD2* which are believed to be involved in the early steps of mycolic acid biosynthesis (Barry et al., 2007), were upregulated in the vitamin C model of dormancy. The KasB protein also involved in mycolic acid biosynthesis was upregulated during dormancy in at least two studies (Starck et al., 2004; Gopinath et al., 2015), but downregulated during potassium depletion (Salina et al., 2014). While some genes associated with fatty acid degradation were upregulated in the vitamin C model, others were downregulated. These contradictions may be attributed to the use of different *in vitro* models of dormancy, different time points of sampling of bacteria and methods with differing sensitivities for analysis of gene or protein expression. It must also be mentioned that a considerably large proportion of the observations of omics studies, await validation.

Areas for further investigation include understanding the differential expression of various Esx secretion systems members, chaperones, proteases and peptidases, during dormancy and reactivation, making these areas for future exploration. While the differential regulation of components of toxin/antitoxin modules have been reported, their significance remains unclear.

Overall, the better we understand dormancy and reactivation at a system level, the better will be the possibility of targeting these processes, and possibly shortening treatment regimens. As an example, omics approaches have unveiled a likely role of the transcriptional regulator ClgR in dormancy as well as in resuscitation. Further studies are required to understand the ClgR regulon and its role in dormancy and resuscitation. There is also an urgent need to develop better *in vivo* models for understanding dormancy and reactivation, so that the end goal of successfully containing TB is achieved in the shortest possible time.

## Author Contributions

JB and MK reviewed the literature and wrote the manuscript. Both authors contributed to the article and approved the submitted version.

## Conflict of Interest

The authors declare that the research was conducted in the absence of any commercial or financial relationships that could be construed as a potential conflict of interest.
